# Association between body fat percentage and urinary flow rate in US adults: analysis of the National Health and Nutrition Examination Survey 2011–2018

**DOI:** 10.1017/S000711452500039X

**Published:** 2025-04-14

**Authors:** Xiao Jin, QiWu Mi, RuJun Mo, ZhaoHua Ye, ShaoWei Fang, JieXin Luo, ZhengGuo Cao

**Affiliations:** The Tenth Affiliated Hospital of Southern Medical University (Dongguan People’s Hospital), Department of Urology, 523000 No.3 South Wanlu Road, Xingu Yong, Wanjiang District, Dongguan City, Guangdong Province, China

**Keywords:** Total body fat percentage, Trunk fat percentage, Urinary flow rate, National Health and Nutrition Examination Survey, Obesity

## Abstract

This study examined the relationship between total body fat percentage (TBFP), trunk fat percentage (TFP), and urinary flow rate (UFR) using NHANES data (2011–2018) from 10 521 participants aged 18–59. Multivariable-adjusted regression models assessed associations between dual-energy X-ray absorptiometry-measured fat percentages and UFR. Results showed that increased TBFP and TFP were inversely associated with UFR (TBFP: *β* = −0·013, 95 % CI [–0·018, −0·007]; TFP: *β* = –0·014, 95 % CI [–0·018, −0·009]). Stratified analyses revealed gender differences: in males, higher TBFP and TFP correlated with lower UFR (TBFP: *β* = –0·011, 95 % CI [−0·019, −0·004]; TFP: *β* = –0·016, 95 % CI [−0·022, −0·009]), but this association was absent in males aged 18–35. In females, both TBFP and TFP were consistently linked to reduced UFR across all age groups (TBFP: *β* = −0·016, 95 % CI [−0·023, −0·008]; TFP: *β* = −0·012, 95 % CI [−0·019, −0·006]). These findings suggest that body and trunk fat accumulation negatively impact UFR, with stronger effects in females and age-dependent variations in males. Further research should explore mechanisms behind these disparities to guide targeted interventions.

The current obesity epidemic poses significant public health challenges due to the increased risk of various chronic diseases associated with obesity. Some studies have identified metabolic syndrome and obesity as important factors exacerbating benign prostatic hyperplasia/lower urinary tract symptoms. The prostate volume can directly affect the urinary flow rate (UFR). However, research on the relationship between obesity and the urinary system has primarily focused on anthropometric measurements such as body weight, BMI and waist circumference^(1–3)^. The impact of obesity on the urinary system remains debated. For example, Seung-Guk Park’s study of 6389 Asian men aged 30–79 found no significant link between BMI and prostate volume after adjusting for visceral fat (trend *P* = 0·152). In contrast, Jin-Ho Park’s research involving 3593 Korean men of the same age range identified a positive correlation between obesity and prostate volume, with obesity defined as BMI ≥ 25 or waist circumference ≥ 90 cm^(4,5)^. This discrepancy highlights the limitations of BMI and similar indicators, which fail to distinguish between fat and non-fat tissues, potentially affecting the accuracy of research findings^(6,7)^. Percentage of body fat represents the proportion of fat within the total body weight. Some studies suggest that indicators such as fat content may be more informative in revealing the relationship between obesity and health outcomes^(8,9)^.

In this study, the UFR refers to the rate at which urine is produced and expelled from the urinary system, calculated by measuring the volume of urine expelled over a specific time period. It reflects the processes of urine generation, transport, storage and expulsion and is influenced by multiple factors of the urinary system, including kidney filtration function, renal pelvis and ureter transport efficiency and urethral voiding capability and also indirectly reflecting bladder function and status^(10–14)^. Therefore, it is an important parameter for assessing bladder function and urinary system health. By measuring both the flow rate and volume of urine, UFR can provide insights into the state of the urinary system and also reflect the body’s hydration status. Maintaining an appropriate UFR is crucial, as a decrease in UFR has been shown to be associated with various urinary system conditions, such as kidney stones, proteinuria and contrast-induced acute kidney injury^(15,16)^.

Dual-energy X-ray absorptiometry (DXA) scanning is a widely accepted and effective method for estimating various body fat parameters, including total body fat mass and truncal fat mass^(1)^. Using this technology, we can directly measure body fat content, thereby avoiding the limitations of traditional obesity indicators that cannot effectively differentiate between muscle and fat content. There has been no direct evidence linking body fat percentage and UFR. In this study, we utilised DXA-based fat mass measurements and analysed the National Health and Nutrition Examination Survey (NHANES) dataset from 2009 to 2018 to explore the relationship between TBFP, TFP and UFR. We hypothesise that the proportions of total body fat and trunk fat are negatively associated with UFR. Additionally, previous studies have indicated that UFR increases with age until around 60 years, and there are significant differences in UFR between males and females^(17)^. To better understand the impact of body fat percentage on UFR in different population groups and guide body fat management strategies, we conducted stratified analyses by gender and age. This research aims to further elucidate the associations of obesity and UFR, which could facilitate early identification of high-risk populations and help reduce the incidence of urinary system disease.

## Materials and methods

### Study population

The NHANES is a representative survey of the US national population that uses a complex, multi-stage, probability sampling design, providing information on nutrition and health for the general US population. The NHANES website provides the detailed questionnaire survey tools, programme manuals, brochures and consent documents for 2011–2018 NHANES. Given the physiological differences between minors and adults, this study excluded individuals under the age of 18. Additionally, since NHANES only collects body fat data from participants under 59 years of age, this study did not include individuals over 59 years old.

Our study analysis was based on data from 2011 to 2018, representing four cycles of NHANES, and excluded 11 532 cases with missing UFR data, 13 467 cases with missing whole body and trunk body fat percentage data and 3636 cases under 18 years of age. A total of 10 521 participants for participants aged 18–59 were included in this study.

The outcome variable is the UFR. The laboratory method for determining UFR was based on a standard protocol^(18)^. UFR is a non-invasive screening tool used to assess and quantify urinary conditions. Participants were asked to record the time of their last urination. The calculation method involves dividing the total volume of collected urine by the time elapsed from the previous urination to the end of the current urination. The specific formula is UFR = (total urine volume)/(total time duration) (ml/min). Comprehensive guidelines for urine collection and handling can be found in the NHANES Laboratory Procedures Manual. To meet acceptability and repeatability standards, each participant could have up to three UFR measurements, and the final average UFR is based on the total number of samples.

The exposure variables are total body fat percentage (TBFP) and trunk fat percentage (TFP), measured using whole-body DXA scanning. Whole-body DXA scans were conducted during four consecutive research periods from 2011 to 2018. Each DXA scan was reviewed and analysed by the Radiology Department of the University of California, San Francisco, which provided body composition data. These data include percentage of body fat (%), fat mass (including head, limbs and trunk areas) and trunk weight (in kilograms). The participant criteria for DXA scanning in NHANES were as follows: individuals aged between 8 and 59 years were eligible. Exclusion criteria include pregnancy (positive urine pregnancy test and/or self-reported pregnancy during the DXA scan), self-reported use of radiographic contrast material (e.g. barium) in the past 7 d and self-reported weight exceeding 450 pounds or height exceeding 6 feet 5 inches (limitations of the DXA equipment).

Based on previous studies using the NHANES database related to UFR, obesity and urinary system diseases, and the KDOQI US Commentary on the 2012 KDIGO Clinical Practice Guideline for the Evaluation and Management of Chronic Kidney Disease^(17,19,20)^, the following variables were included in our analysis as covariates: age, sex, race (non-Hispanic white, non-Hispanic black, Hispanic and other), family poverty ratio (defined as the annual income compared to the federal poverty line and categorised as < 1·3, 1·3–3·5 and > 3·5, classification follows the recommendations from the Supplemental Nutrition Assistance Programme regarding the family poverty ratio, with categories as follows: < 1·3 for extremely low-income households,1·3–3·5 for low to moderate-income households and ≥ 3·5 for middle to high-income households. For more details, please refer to www.cdc.gov/nchs/nhanes/, and education (less than high school, high school and above high school). Based on the question ‘Do you now smoke cigarettes?’, smoking status was categorised as ‘Yes’ and ‘No’. BMI was also collected. Self-reported medical conditions included hypertension (yes/no), hypercholesterolaemia (yes/no), cancer (yes/no), diabetes (yes/no), CVD (yes/no) and gout (yes/no). Detailed information can be publicly accessed at http://www.cdc.gov/nchs/nhanes/.

### Statistical analysis

We conducted weighted and variance estimation analysis on the dataset to identify significant differences. We used a weighted multivariable linear regression model to assess the association between body fat percentage and urine flow rate. For categorical variables, we employed weighted chi-square tests, and for continuous variables, we used weighted linear regression models to calculate differences between groups. The weights used in the NHANES survey are designed to adjust the importance of variables to more accurately reflect their impact on outcomes. According to the NHANES website, we utilised the weight code ‘wtmec4yr’. To better understand the impact of body fat percentage on UFR in different demographic groups and to guide body fat management plans for various populations, considering gender and age differences in lower urinary tract structures, we conducted stratified analyses by gender and age. Since physiological differences exist between minors and adults, minors were excluded from this study. Additionally, due to NHANES’s inclusion criteria for body fat percentage data being restricted to individuals under 59 years old, individuals over 59 were excluded. Based on previous studies^(21)^, we divided age into two groups: 18–35 years and 36–59 years. Subgroup analysis was also performed using stratified multifactor regression analysis. To address the nonlinear relationship between body fat percentage and urine flow rate, we applied smoothing curve fitting methods. All analyses were conducted using the R software package (http://www.Rproject.org) and licensed statistical data (http://www.empowerstats.com). For statistical significance, a *P*-value of less than 0·05 was considered significant.

## Result

### Research population characteristics

Our study included a total of 10 521 people from NHANES from 2011 to 2018, of whom 5265 were male and 5256 were female. The characteristics of our study population were listed in Table 1 and were representative of the entire country after weighting. The age range was 18–59 years old, with an average age of 35·69 (±13·14) for males and 36·32 (±13·16) for females. The average BMI was 27·90 (±6·04) for males and 28·67 (±7·44) for females. In terms of medical history, except for a history of diabetes, stroke and hypertension, which showed no statistically significant differences between the two groups, there were significant differences in the history of gout, CHD, cancer, high cholesterol and smoking. In females, the average TBFP was 38·37 % (±6·33), and the average TFP was 36·12% (±7·73). In males, the average TBFP was 26·47% (±6·28), and the average TFP was 26·55% (±7·51).

**Table 1.** Socio-demographic and lifestyle characteristics of NHANES participants (2011–2018)^*^ by gender (Mean values and standard deviations; Numbers and percentages)

	Total (10 521)				*P*-value
	Male (*n* 5265)		Female (*n* 5256)		
	Means	sd	Means	sd	
Continuous variables, means (sd)					
Age (year)	35·69	13·14	36·32	13·16	0·01
BMI (kg/m^2^)	27·90	6·04	28·67	7·44	0·01
Total fat percentage	26·47	6·28	38·37	6·33	< 0·001
Trunk fat percentage	26·55	7·51	36·12	7·73	< 0·001
Urine flow rate (ml/min)	1·11	1·12	1·02	0·92	< 0·001
	*n*	%	*n*	%	
Categorical variables, *n* (%)					
Race					0·02
Mexican American	809	15·36	730	13·88	
Other Hispanic	447	8·49	392	7·45	
Non-Hispanic White	2029	38·53	2059	39·17	
Non-Hispanic Black	1223	23·22	1310	24·92	
Other Race -including multi-racial	757	14·37	765	14·55	
Education					0·017
< High school	579	10·99	522	9·93	
High school	550	10·44	478	9·09	
> High school	1869	35·49	1892	35·99	
Family poverty ratio					0·013
< 1·3	1517	28·81	1568	29·83	
1·3–< 3·5	1878	35·67	1955	37·19	
> 3·5	1371	26·04	1316	25·03	
Past history (yes)					
Diabetes	328	6·23	332	6·31	0·595
Stroke	54	1·02	61	1·16	0·795
Hypertension	1077	20·45	1042	19·82	0·655
Hypercholesterol	1168	22·18	1037	19·73	0·007
Gout	146	2·27	60	1·14	< 0·001
CHD	58	1·10	21	0·40	< 0·001
Cancer	109	2·44	211	4·72	< 0·001
Smoke	2125	40·36	1425	27·11	< 0·001

NHANES, National Health and Nutrition Examination Survey.

Family poverty ratio: < 1·3 for extremely low-income households; 1·3–3·5 for low to moderate-income households and ≥ 3·5 for middle to high-income households.

* Sample size was weighted to be nationally representative.

### Body fat percentage and urine flow rate

As shown in Table 2, in the 18–59-year-old population, an increase in TBFP was inversely associated with UFR (*β* = –0·013, 95 % CI –0·018, −0·007), with similar magnitude observed in the age group of 36–59 (*β* = –0·014, 95 % CI –0·022, −0·007). An increase in TFP was also inversely associated with UFR (*β* = –0·014, 95 % CI –0·018, −0·009), with the 36–59-year-old age group showing even stronger significance (*β* = –0·017, 95 % CI –0·023, −0·011).

**Table 2.** Association of total and trunk fat percentage with urine flow rate (UFR) in US adults by age group

	All ages		Stratified by age groups				*P*-value^*^
	18–59 years		18–35 years (*n* 4982)		36–59 years (*n* 5539)		
	*β*	95 % CI	*β*	95 % CI	*β*	95 % CI	
Total fat percentage							
Model 1^†^	–0·008	–0·010, −0·005	–0·008	–0·011, −0·005	–0·007	–0·010, –0·004	< 0·001
Model 2^‡^	–0·009	–0·012, −0·006	–0·006	–0·010, −0·002	–0·013	–0·018, –0·008	< 0·001
Model 3^§^	–0·013	–0·018, −0·007	–0·010	–0·018, −0·002	–0·014	–0·022, –0·007	< 0·001
Trunk fat percentage							
Model 1^†^	–0·008	–0·011, −0·006	–0·007	–0·010, −0·004	–0·010	–0·013, −0·006	< 0·001
Model 2^‡^	–0·009	–0·011, −0·006	–0·005	–0·008, −0·001	–0·013	–0·017, −0·009	< 0·001
Model 3^§^	–0·014	–0·018, −0·009	–0·009	–0·016, −0·002	–0·017	–0·023, −0·011	< 0·001

* Cross–product interaction terms (fat percentage × age) were used to test the interaction effect of age and fat distribution on UFR.

† Model 1 adjust for none.

‡ Model 2 adjust for race and gender.

§ Model 3 adjust for race, gender, education, family poverty ratio, diabetes, stroke, hypertension, hypercholesterol, gout, CHD, cancer, smoke and BMI.

**Figure 1.**
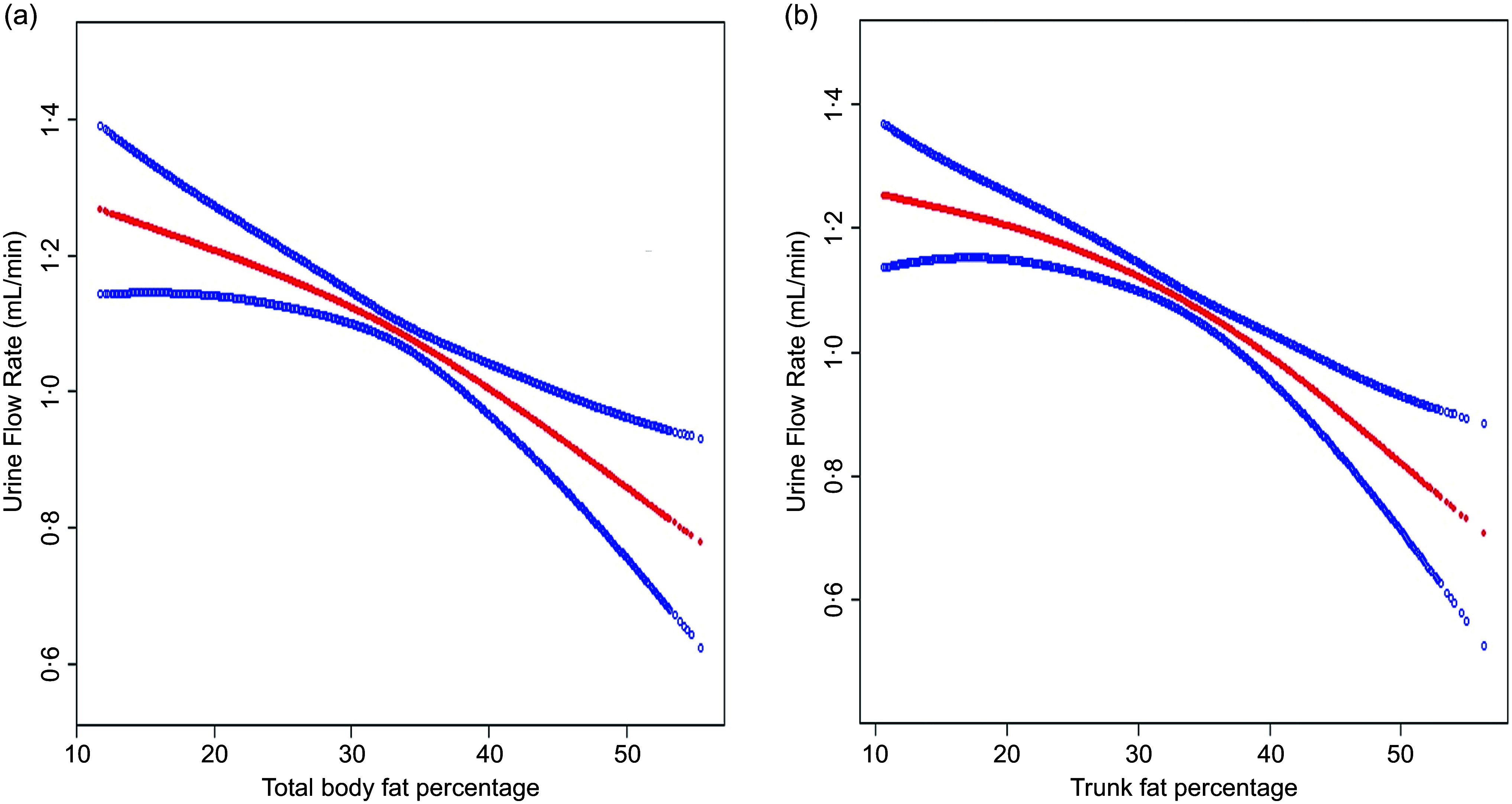
Smooth curve fitting between body fat percentage and urinary flow rate. The model was adjusted for race, education, family poverty ratio, diabetes, stroke, hypertension, hypercholesterolaemia, gout, CHD, cancer, smoking and BMI. (a) Smooth curve fitting between total body fat percentage and urinary flow rate. (b) Smooth curve fitting between trunk fat percentage and urinary flow rate.

We also stratified the study sample by gender (Table 3). A negative association with UFR was observed in both males (TBFP: *β* = –0·011, 95 % CI −0·019, −0·004; TFP: *β* = –0·016, 95 % CI −0·022, −0·009) and females (TBFP: *β* = –0·016, 95 % CI −0·023, −0·008; TFP: *β* = –0·012, 95 % CI −0·019, −0·006). Notably, this association was not significant in the male group aged 18–35 (TBFP: *β* = –0·005, 95 % CI −0·015, 0·006; TFP: *β* = –0·008, 95 % CI −0·017, 0·002). The smooth curve fitting of TBFP and TFP with UFR is shown in Figure 1.

**Table 3.** Association of total and trunk fat percentage with urine flow rate in US adults by gender and age group, NHANES 2011–2018

	All ages		Stratified by age groups				*P*-value^*^
	18–59 years		18–35 years (*n* 4982)		36–59 years (*n* 5539)		
	*β*	95 % CI	*β*	95 % CI	*β*	95 % CI	
Male							
Total fat percentage							
Model 1^†^	–0·005	–0·009, 0·000	–0·005	–0·011, 0·001	–0·004	–0·011, 0·004	0·068
Model 2^‡^	–0·005	–0·009, 0·000	–0·005	–0·011, 0·002	–0·004	–0·012, 0·004	0·062
Model 3^§^	–0·011	–0·019, –0·004	–0·005	–0·015, 0·006	–0·015	–0·025, –0·004	0·002
Trunk fat percentage							
Model 1^†^	–0·005	–0·010, –0·001	–0·005	–0·010, 0·001	–0·007	–0·013, 0·000	0·010
Model 2^‡^	–0·005	–0·010, –0·001	–0·004	–0·010, 0·001	–0·007	–0·013, 0·000	0·011
Model 3^§^	–0·016	–0·022, –0·009	–0·008	–0·017, 0·002	–0·019	–0·029, –0·010	< 0·001
Female							
Total fat percentage							
Model 1^†^	–0·013	–0·017, –0·009	–0·007	–0·012, –0·001	–0·020	–0·026, –0·014	< 0·001
Model 2^‡^	–0·013	–0·018, –0·009	–0·008	–0·013, –0·002	–0·020	–0·026, –0·013	< 0·001
Model 3^§^	–0·016	–0·023, –0·008	–0·019	–0·031, –0·007	–0·014	–0·024, –0·005	< 0·001
Trunk fat percentage							
Model 1^†^	–0·011	–0·015, –0·008	–0·005	–0·009, 0·000	–0·018	–0·023, –0·013	< 0·001
Model 2^‡^	–0·011	–0·015, –0·008	–0·005	–0·010, –0·001	–0·018	–0·023, –0·012	< 0·001
Model 3^§^	–0·012	–0·019, –0·006	–0·012	–0·022, –0·002	–0·014	–0·022, –0·006	< 0·001

* Cross–product interaction terms (fat percentage × age) were used to test the interaction effect of age and fat distribution on UFR.

† Adjust for none.

‡ Adjust for race.

§ Adjust for race, education, family poverty ratio, diabetes, stroke, hypertension, hypercholesterol, gout, CHD, cancer, smoke and BMI.

## Discussion

Our study is the first nationally representative US population study analysing the association between body fat percentage and UFR. After adjusting for the association between TBFP and TFP with UFR, we found that both were related to UFR in US adults. Our research shows that in a fully adjusted model, a 1 % increase in TBFP is associated with a 0·013 ml/min decrease in UFR, and a 1 % increase in TFP is associated with a 0·014ml/min decrease in UFR. In stratified analyses by different populations, this negative association is prevalent among adult women. In men, considering potential influencing factors such as activity level, testosterone levels or prostate function, the negative association between total and TFP and UFR was not significant among men aged 18–35. However, in middle-aged men, the accumulation of both total and trunk fat may negatively impact UFR. These results provide strong evidence that the accumulation of body fat negatively affects urinary system function.

Maintaining an appropriate UFR is crucial, as a decline in UFR has been linked to various urinary system disorders, such as kidney stones, proteinuria and contrast-induced acute kidney injury^(15,16)^. Previous research on the relationship between obesity and the urinary system has primarily focused on anthropometric parameters such as weight, BMI and waist circumference. For example, Seung-Guk Park and colleagues studied 6389 Asian men aged 30–79 and found that after adjusting for visceral fat tissue, BMI was not related to prostate volume (trend *P* = 0·152). However, visceral fat tissue remained positively correlated with prostate volume even after adjusting for BMI (trend *P* = 0·005). Similarly, Jin-Ho Park and colleagues studied 3593 Korean men aged 30–79 and found that obesity was positively correlated with prostate volume. Notably, in this study, obesity was defined as BMI ≥ 25 or waist circumference ≥ 90 cm^(4,5)^. Previous studies have shown that the relationship between these parameters and the urinary system is not yet consistent, possibly because the main limitation of these metrics is their inability to differentiate between body fat and muscle mass^(22,23)^. In fact, the paradox of traditional obesity metrics is also present in research on other systemic diseases. For instance, Yousung Park’s study indicates that over the past decade, the BMI range with the lowest CVD mortality rate has shifted from 23–25 kg/m^2^ (overweight) to 25–29·9 kg/m² (moderate obesity). This suggests that obese individuals have a lower all-cause cardiovascular mortality rate compared to those with normal weight, which is clearly unreasonable and indicates that BMI is a limited measure of true obesity^(24)^. We believe that the primary impact of obesity on the body is due to the accumulation of fat. Therefore, our study has moved away from commonly used traditional obesity metrics and opted to use whole-body DXA scanning to measure body fat percentage, thereby obtaining more accurate results. Compared to traditional BMI, body fat percentage better reflects the impact of fat on the body. Our research found that, even after adjusting for confounding factors, there is an overall negative association between UFR and both TBFP and TFP, for both men and women. Thus, we conclude that body fat content has a negative effect on UFR.

In males aged 18–35, the association between TBFP and UFR was not significant. Similarly, the association between TFP and UFR was not significant. But in women aged 18–35, this phenomenon was not observed. This suggests that in young men, body fat percentage does not have a significant impact on UFR. Potential factors such as daily activity level, testosterone levels and prostate function might influence this relationship.

In males aged 36–59, the increase in TBFP was significantly associated with a decrease in UFR, which is consistent with the overall trend in the population. The association between TFP and UFR was also significant. This indicates that in middle-aged men, both total and trunk fat accumulation may negatively impact urinary function, with trunk fat showing a more pronounced negative effect.

In fact, the potential mechanisms by which body fat affects urine flow rate are currently unclear. First, the development of benign prostatic hyperplasia in men with high body fat percentage may be associated with increased aromatase activity, which is expressed in fat cells and converts testosterone into estradiol^(25)^. Elevated oestrogen levels activate the prostate’s ER-*α* receptors and lead to prostatic hyperplasia.

Other studies have shown that excess fat can lead to a reduction in UFR and even urinary voiding dysfunction by inducing fibrosis in the prostate and urethral tissues^(26)^. Fat factors, such as interleukins, CC- and CXC-type chemokines and TGF-*β*, are believed to play a role in this process^(27,28)^. Regarding the impact of fat on the urinary system, the protein fetuin-A has attracted attention due to its strong induction of pro-inflammatory signals in adipose tissue. Fetuin-A affects renal blood flow by acting on the perivascular fat around the renal sinus and potentially on endothelial cells, which may directly influence kidney function^(29)^. In summary, these studies suggest that there is an association between fat and a reduction in UFR, likely due to chronic inflammation^(30–32)^.

Trunk fat reflects visceral fat and can predict metabolic status better than BMI. Current research often uses waist circumference or waist-to-hip ratio to indirectly represent trunk obesity. For example, the National Cholesterol Education Program Adult Treatment Panel III defines metabolic syndrome based on waist circumference measurement rather than BMI^(33)^. Research has shown that trunk obesity is more strongly associated with adverse CVD outcomes and mortality than BMI^(34)^. Additionally, research has shown that visceral fat tissue measured by DXA scanning is more strongly associated with adverse cardiovascular outcomes and mortality compared to waist-to-hip ratio or waist circumference^(35)^. This also demonstrates the advantages of whole-body DXA scanning. Fat accumulation, particularly in the trunk region, may exert direct mechanical pressure on the urinary system. Studies indicate that fat accumulation in the abdominal and pelvic areas may exert additional pressure on the bladder and, over time, stress the ligaments and nerves, leading to pelvic floor dysfunction^(36)^. This pressure can reduce the effective capacity of the bladder and affect its urinary function, leading to a decreased urine flow rate. This effect is particularly pronounced in middle-aged individuals, as body fat in this age group tends to accumulate more in the trunk area. This is similar to the results of our study.

While delving into the relationship between body fat percentage and UFR, we should also consider other factors that may influence this relationship. For instance, dietary habits and lifestyle may have varying effects on UFR across different age groups. Studies have shown that high sugar and fat intake, as well as high salt consumption, are associated with lower UFR, which may partially explain the correlation between obesity and UFR^(37)^. Additionally, physical activity and exercise levels are recognised as factors influencing UFR, and these factors may vary across different age groups^(17)^. This could be one of the potential reasons for the lack of a significant correlation between body fat percentage and urine flow rate in the 18–35 age group in this study.

Our study is the first nationwide representative study in the USA to analyse the association between body fat percentage and UFR. Its strength lies in using data from a large and representative cross-sectional survey, and importantly, employing DXA whole-body scans to measure body fat percentage, which avoids the limitations of traditional obesity metrics that fail to accurately differentiate body composition. It also provides the first evidence of an association between body fat percentage and UFR and quantifies the impact of body fat percentage on UFR. However, there are some limitations. First, it is a cross-sectional study, meaning it only represents participants’ status at the time of testing, and causality cannot be inferred. Further prospective studies are needed to support definitive conclusions. Second, the UFR data available in the NHANES database do not include peak urine flow rate or bladder contraction capacity, which directly reflects bladder contraction capability. Despite this, the average UFR data provided by NHANES can still serve as an effective reference for assessing bladder function^(10,11)^. Additionally, there are potential confounding factors that may still have an impact. Future research could incorporate these confounding factors into the analysis model for a more accurate assessment of the relationship between body fat percentage and UFR.

### Conclusion

Our study indicates that in the population aged 18–59 years, both total and truncal fat accumulation are significantly negatively associated with urine flow rate. This negative association is not significant among males aged 18–35 years. Maintaining an appropriate body fat percentage may help preserve the urine flow rate. Future research should explore the mechanisms behind these gender and age differences to provide more precise evidence for developing targeted prevention and intervention measures.
